# A Virtual Reality-Based Multimodal Approach to Diagnosing Panic Disorder and Agoraphobia Using Physiological Measures: A Machine Learning Study

**DOI:** 10.3390/diagnostics15172239

**Published:** 2025-09-03

**Authors:** Han Wool Jung, Hyun Park, Seon-Woo Lee, Ki Won Jang, Sangkyu Nam, Jong Sub Lee, Moo Eob Ahn, Sang-Kyu Lee, Yeo Jin Kim, Daeyoung Roh

**Affiliations:** 1 Department of Psychiatry, Yongin Severance Hospital, Yonsei University College of Medicine, Yongin 16995, Republic of Korea; jhw2@naver.com; 2 Department of Integrative Medicine, Yonsei University College of Medicine, Seoul 06229, Republic of Korea; 3 Division of Software, Hallym University, Chuncheon 24252, Republic of Korea; 4 Mind-Neuromodulation Laboratory, Hallym University College of Medicine, Chuncheon 24253, Republic of Korea; 5 Department of Psychology, Hallym University, Chuncheon 24252, Republic of Korea; 6 Department of Emergency Medicine, Hallym University College of Medicine, Chuncheon 24253, Republic of Korea; 7 Department of Psychiatry, Hallym University College of Medicine, Chuncheon 24253, Republic of Korea; 8 Department of Neurology, Kangdong Sacred Heart Hospital, Seoul 05355, Republic of Korea

**Keywords:** biomarkers, digital markers, virtual reality assessment, heart rate variability, skin conductance, galvanic skin response, electrodermal activity

## Abstract

**Objectives**: Virtual reality (VR) has emerged as a promising tool for assessing anxiety-related disorders through immersive exposure and physiological monitoring. This study aimed to evaluate whether multimodal data, including heart rate variability (HRV), skin conductance response (SCR), and self-reported anxiety, collected during VR exposure could classify patients with panic disorder and agoraphobia using machine learning models. **Methods**: Seventy-six participants (38 patients with panic disorder and agoraphobia, 38 healthy controls) completed 295 total VR exposure sessions. Each session involved two road and two supermarket scenarios designed to induce anxiety. Inside the sessions, self-reported anxiety was measured along with physiological signals recorded by photoplethysmography and SCR sensors. HRV measures of heart rate, standard deviation of normal-to-normal intervals, and low-frequency to high-frequency ratio were extracted along with SCR peak frequency and average amplitude. These features were analyzed using Gaussian Naïve Bayes (GNB), k-Nearest Neighbors (k-NN), Logistic Ridge Regression (LRR), C-Support Vector Machine (SVC), Random Forest (RF), and Stochastic Gradient Boosting (SGB) classifiers. **Results**: The best model achieved an accuracy of 0.83. Most models showed specificity and precision ≥0.80, while sensitivity varied across models, with several reaching ≥0.82. Performance was stable across major hyperparameters, VR-stimulus settings, and medication status. The patients reported higher subjective anxiety but exhibited blunted physiological responses, particularly in SCR amplitude. Self-reported anxiety demonstrated higher feature importance scores compared to other physiological properties. **Conclusion**: VR exposure with self-reported anxiety and physiological measures may serve as a feasible diagnostic aid for panic disorder and agoraphobia. Further refinement is needed to improve sensitivity and clinical applicability.

## 1. Introduction

Virtual reality (VR) is increasingly being explored as a diagnostic and measurement tool for patients with psychiatric disorders. VR allows and assists clinicians to evaluate symptoms, identify symptom-related markers, establish factors that can predict disorders, and verify the putative cause of the disorders [1]. Therefore, numerous attempts to utilize VR for the assessment of various anxiety disorders have been made, including social anxiety disorder, posttraumatic stress disorder, and phobic disorders [1,2]. There have also been attempts to adopt VR for the assessment of panic disorder and agoraphobia by evaluating patients’ anxiety levels during virtual exposure to anxiety-inducing environments or panic attacks. Several notable studies have aimed to incorporate VR exposure as a multimodal assessment tool for stress or panic symptoms to overcome the limitations of traditional assessments that predominantly depend on interviews or self-reports. Ahilan et al. (2023) developed a VR-based embedded system using electromyography, pulse oximetry, and galvanic skin response (GSR) sensors, showing its potential to diagnose users’ stress levels during VR exposure [3]. Kim et al. (2023) combined VR exposure with physiological markers such as photoplethysmography (PPG) or heart rate variability (HRV) as well as self-reported anxiety to assess panic symptoms, which is based on the multimodal approach that adopts various physiological benchmarks for the diagnosis and assessment of mental disorders [4]. These approaches are considered a breakaway from the traditional subjective diagnostic methods [5,6].

Although these studies demonstrated the potential of VR as an assessment tool for panic disorder, they also reveal certain limitations. First, the current approaches still seem immature to be considered a complete multimodal assessment tool. For instance, Kim et al. (2023) incorporated HRV data with self-reported anxiety during VR sessions to diagnose panic disorder, but only reported mere significance levels of each variable without the combined use of various HRV features with self-reports [4]. In contrast, Ahilan et al. (2023) adopted machine learning for comprehensive stress prediction utilizing all the acquired data; however, due to the limitation of the neural network model, the individual predictive ability of each feature was not provided, and its medical significance remains unclear [3]. Determining medical significance is essential because increased anxiety levels during VR sessions may not be a primary marker for panic disorder. To illustrate, Kim et al. (2023) observed that both self-rated and physiological anxiety levels were mostly not significantly associated with the Panic Disorder Severity Scale (PDSS), which is considered a gold standard for panic disorder, but instead correlated with the Generalized Anxiety Disorder-7, a gold standard for generalized anxiety [4]. To identify whether these variables are relevant markers for panic disorder, we need to meticulously verify whether the variables can adequately predict the symptoms of the patients, beyond mere correlational or “black-box” reports.

Therefore, medical diagnosis of mental disorders using VR and psychophysiology should satisfy three criteria: (i) aggregate features to develop a comprehensive predictive model; (ii) demonstrate that the measures included in the model can contribute to the overall performance; and (iii) adopt the criteria of what they test that are medically valid. Park et al. (2025) developed a multimodal model to predict social anxiety disorder using acoustic data and physiological signals such as heart rate (HR) and GSR collected during VR sessions, achieving an area under the receiver operating characteristic curve scores of up to 0.866 [7]. A similar attempt was made by Tsai et al. (2022) [8], although they did not adopt VR methods. They tried to predict future panic attacks of the patients with panic disorder by gathering and analyzing not only self-reports but also the physiological factors measured using data obtained from wearable devices, such as HR and sleep durations, and environmental factors such as air quality. Overall, their models demonstrated high performance, achieving an accuracy of up to 0.81 [8]. Similarly, Kim et al. (2023) reported a comprehensive HRV index derived from a pre-equipped device, with effect sizes that were lower than self-reports and only significant for some sessions [4].

These results suggest a need for careful implementation of valid symptom-specific features for panic disorder and agoraphobia. Consequently, we adopted diverse HRV features extracted from the PPG raw signals, which are considered relevant to anxiety disorders [7,9]. Additionally, we measured skin conductance or GSR, which is considered one of the representative physiological properties of patients with panic disorder [10]. For HRV features, HR, the standard deviation of the normal-to-normal interval (SDNN), and the low frequency/high frequency (LF/HF) ratio were considered relevant. For HR, Freire et al. (2010) reported that when they were watching anxiogenic computer simulations, panic and agoraphobia patients with panic attacks exhibited higher HR than healthy people, whereas those without panic attacks showed rather lower HR, despite the lack of overall difference between patients and healthy controls [11]. SDNN indicates the variability of sinus heartbeats and is associated with the physical ability of stress coping [9,12]. The LF/HF ratio reflects the relative dominance between sympathetic and parasympathetic activities, which is considered germane to panic-related anxiety [9,13]. Zhang et al.’s (2020) meta-analysis concluded that patients with panic disorder exhibit short-term increases in LF/HF ratio but long-term decreases in SDNN [12]. For GSR, Freire et al. (2010) reported that while watching the anxiogenic computer simulations, higher tonic skin conductance level (SCL) and the magnitude of phasic skin conductance response (SCR) were observed among patients with panic and agoraphobia compared to healthy people, although the frequency of SCR did not differ [11]. Pruneti et al. (2010), when they were conducting stress response and recovery tasks, reported that SCR values of patients with panic disorder were higher than those with major depressive disorder or obsessive–compulsive disorder [14]. Moreover, a recent computer simulation study by Freire et al. (2020) revealed that patients with panic attacks tended to exhibit increased SCL over the time of exposure, whereas patients without panic attacks tended to demonstrate decreased SCL during the exposure sessions like healthy people [15].

In summary, we aimed to evaluate the accuracy of a multimodal physiological measurement VR exposure program in distinguishing between agoraphobic patients and healthy individuals by analyzing HRV, GSR, and self-reported anxiety. To this end, we employed various machine learning models to classify patients with panic disorder and agoraphobia. Also, beyond reporting overall performance and individual feature importance, we focused on enhancing the models’ explainability, aiming to improve the clinical applicability of the models. We also focused on the robustness of the results across diverse VR stimuli, hyperparameter settings, and medication status to see the potential generalizability of the models.

## 2. Materials and Methods

### 2.1. Participants

The participants were recruited through online advertisements and from outpatient clinics at a local hospital. Inclusion criteria for patients required a primary diagnosis of either panic disorder or agoraphobia according to the Diagnostic and Statistical Manual of Mental Disorders, Fifth Edition (DSM-5). Healthy controls were required to have no current mental disorders. For both groups, eligibility criteria included 18 to 65 years of age and the absence of cognition and movements or motor impairments that could hinder participation in the VR tasks or completion of questionnaires. Exclusion criteria included a history of epileptic or photosensitive seizures, suicide attempts within six months, and serious psychiatric disorders such as psychosis or bipolar I disorder. All assessments were conducted by a researcher with a master’s degree in psychology under the supervision of a board-certified psychiatrist. This study was approved by the Institutional Review Board, and all participants provided written informed consent.

### 2.2. Implementation of VR Exposure

**Figure 1:**
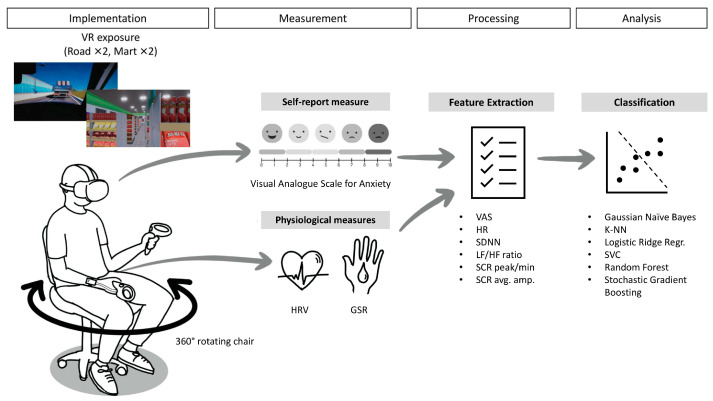
Visualized outline of the current study.

Figure 1 illustrates the overall study procedure from implementation to analysis. In general, each participant was exposed to a total of four VR sessions (two road and two mart sessions) that may induce agoraphobic anxiety in individuals with panic disorder and agoraphobia. The road and mart sessions in VR were designed to simulate environments that typically induce significant agoraphobic anxiety, specifically: (1) using public transportation and (2) crowds and public spaces. The validity of the simple prototype of the simulated environments used in this study was demonstrated in our previous research [16]. During the road sessions, participants had to sit in the passenger seat and pass through a highway, bridge, and tunnel in the first session. However, in the second car session, either the bridge or tunnel subsection was omitted, and instead, a random event that may strain the participants occurred, such as thick fog or road construction. During the mart sessions, the participants had to buy some ingredients with a shopping cart at a grocery store. In the first session, participants were tasked with buying two designated ingredients, and in the second session, they needed to buy one ingredient. The cart automatically drove itself to the target, but the participants had to actively pick up the ingredients and put them into the cart. Like the road session, the second mart session included a random event that may strain the participants, such as other customers blocking the way or a supermarket staff member initiating a conversation with the participants. For both sessions, voice instructions were provided on VR usage and coping strategies for managing anxiety during the exposure sessions. Over the sessions, the crowd density (the density of cars or other customers), brightness (midday, day, night, or midnight during the car sessions; the lucidity of indoor lights during the mart sessions), and spatial dimensions (the overall size of the supermarket, height of the ceiling, and width of the aisle) were personalized for each participant. The personalized settings were based on the pre-exposure survey responses about the degree to which they feel anxious (i) when in crowded places versus when alone in a place where no one is around, (ii) when they are in bright versus dark places, and (iii) when they are in wide versus closed spaces. The VR exposure environments used in this study were developed with technical support from nGarden (Daegu, Republic of Korea).

Participants sat in a reclining, stationary chair capable of 360° rotation in a comfortable place and experienced VR scenarios via an Oculus Quest 2 head-mounted display (Meta Platforms Inc., Menlo Park, CA, USA), wirelessly connected to a desktop computer for high-quality graphic rendering. Participants used a right-hand VR controller to perform tasks and self-rated their anxiety levels, while physiological sensors (PPG and GSR) were attached to their left hand. They were asked to put their left hand on the armrest and only use the right hand to manipulate the VR controller. A research staff member monitored the entire process of the three VR scenarios and was expected to respond to patient requests or emergencies. Each session lasted approximately 5 min, with slight variations (~1 min) owing to the difference in the scenario settings and the participants’ response speed. The entire study lasted 20–25 min.

### 2.3. Measurement and Feature Extraction

The participants’ self-reported anxiety levels and physiological properties were measured during the VR sessions. The participants’ anxiety levels were measured during the VR exposure using the Visual Analogue Scale (VAS), an 11-point scale from 0 (not at all anxious) to 10 (overwhelmingly anxious). These self-reported anxiety levels were recorded 2–4 times per session based on the session length; particularly, at the beginning of the session and end of each subsection of the scenario (e.g., when the car passed through the highway/bridge/tunnel or when the participant finished picking up the item). The average anxiety score through the session was saved for analysis.

During the sessions, a PPG sensor and two GSR sensors were affixed to the participant’s left hand, along with a Shimmer3 GSR + Unit (Shimmer Research Ltd., Dublin, Ireland) at a sampling rate of 51.2 Hz. The GSR sensors were attached to the index and middle fingers, and the PPG sensor was attached to the ring finger. The PPG raw signals were processed and analyzed using NeuroKit2 [17]. Finally, the mean HR, SDNN, and LF/HF ratio were extracted for each session. The GSR raw signals were downsampled to 17.07 Hz and smoothed using a Gaussian window of a width of 8. To extract the peaks and amplitudes of the SCR, a Discrete Decomposition Analysis was conducted. This method adopts the nonnegative decomposition introduced by Benedek and Kaernbach (2010), who argued that this analysis is well-applicable to the variations from the standard signals and more sensitive compared to the conventional peak detection method, especially when the inter-stimulus intervals are short [18]. The analysis was conducted with a 0.2 s smooth window and a grid size of 60, and tau values were automatically optimized to minimize the errors. The peaks of the phasic activity were detected with the peak amplitudes (μS) at a significance level of 0.001. Finally, the number of peaks divided by the length of the session (minutes) and the average amplitude of the detected peaks were extracted for each session. The GSR signals were processed and analyzed using Ledalab (http://www.ledalab.de).

### 2.4. Machine Learning Analysis

Utilizing the above measures (VAS score, HR, SDNN, LF/HF ratio, SCR peak, and SCR average amplitude), classification of patients and healthy people was conducted using various machine learning methods. To increase the size of the dataset, each session, rather than each participant, was included as an individual subject for the machine learning analysis. For all the models, the entire dataset was divided into training (80%) and test (20%) sets. For the main analysis, cross-validation was performed using 1000 iterations of the train-test-set splits. The data splits were stratified to maintain the proportion of patients versus healthy controls and the distribution of sessions across VR environments (two roads and two marts). To avoid data leakage, participant-level grouping was enforced, ensuring that sessions from the same participant were not included in both the training and test sets within the same cross-validation loop. All analyses for classification were conducted with scikit-learn [19], and the coding was assisted by ChatGPT (OpenAI, San Francisco, CA, USA).

Machine learning classifiers included Gaussian Naïve Bayes (GNB), k-Nearest Neighbors (k-NN), Logistic Ridge Regression (LRR), *C*-Support Vector Machine (SVC), Random Forest (RF), and Stochastic Gradient Boosting (SGB). For GNB, the prior probabilities were automatically adjusted according to the data. The k-NN model used a brute-force algorithm and Euclidean distance with uniform weight, and various values of the number of neighbors were tested to identify the robustness of the test results. The LRR model used an L2 penalty term (Ridge) with a primal formulation. SVC was based on the Radial Basis Function with the gamma parameter set to scale. For k-NN, LRR, and SVC, the data were normalized before the main analysis. The normalization was performed after the train-test split within each cross-validation loop to prevent data leakage. For both LRR and SVC, the hyperparameter *C* (the inverse strength of regularization) was initially set to 1 as a baseline value and subsequently varied to assess the robustness of the results.

The RF model adopted Gini impurity with a maximum tree depth of 5. The minimum number of samples was restricted to 3 for internal node splits and 2 for leaf node splits. The SGB model adopted a stochastic algorithm that only utilized half of the entire dataset to fit a tree. The maximum depth of the nodes was limited to 4, and employed exponential loss, incorporating AdaBoost-like features as well. For both ensemble models (RF and SGB), the number of estimators (trees/boosting stages) was tested with multiple values to identify the robustness of the test results.

For performance testing, average accuracy, specificity, sensitivity, and precision scores with their 95% confidence intervals (CIs) were calculated by aggregating the scores in the cross-validation loops. To determine the optimal classification threshold, fine-tuning within each cross-validation loop was performed by selecting the best threshold value from the grid [0.25, 0.30, 0.35, 0.40, 0.50] that maximized the F1 score. Results with various hyperparameters (e.g., k for k-NN, *C* for LRR and SVC, and the number of estimators for RF and SGB) were provided to identify the optimal model configurations and test the robustness of the results. Additionally, subgroup analyses were conducted to compare model performance across different VR scenes (Road 1, Road 2, Mart 1, Mart 2), personalized VR settings (brightness and crowd density), and concomitant use of antianxiety or benzodiazepine (BZD) medications. For the analyses, performance metrics were aggregated over 1000 iterations of train-test splits performed within each subgroup. Finally, feature importance for RF and SGB models was determined by aggregating the Shapley Additive Explanation (SHAP) values obtained from the cross-validation loops.

## 3. Results

### 3.1. Participants and Descriptive Statistics

A total of 76 participants (38 patients with panic/agoraphobia and 38 healthy controls) were included in this study. All participants completed four sessions each; however, nine sessions (four from patients and five from healthy controls) lasted less than two minutes and were excluded, resulting in 295 sessions remaining for analysis.

Table 1 summarizes the descriptive statistics and group differences in the main variables. No significant demographic differences were observed between the two groups. The patient group and healthy control group showed significant differences in baseline PDSS scores. The state anxiety subscale of the State-Trait Anxiety Inventory (STAI-S) was higher in patients both before and after VR exposure; however, patients’ STAI-S scores slightly decreased following the exposure, possibly reflecting a habituation effect. Many participants in the patient group were taking psychiatric medications, such as antidepressants or BZDs, whereas none of the healthy control group reported current psychiatric medication use. Overall, the sessions of patients reported significantly higher VAS scores and SDNN but lower SCR amplitude than the sessions of the healthy controls. Also, the patient sessions tended to have numerically higher LF/HF ratio and lower SCR peak rate than the healthy control sessions, although these differences were not statistically significant. Overall, the patients demonstrated stronger stress reactions as observed in self-reported anxiety and HRV features but exhibited blunter reactions in skin responses.

**Figure 2:**
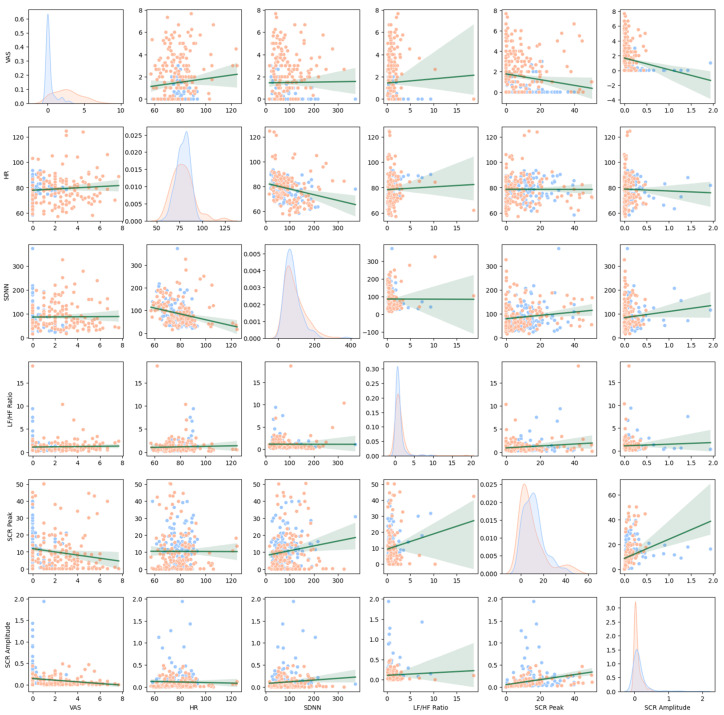
The correlogram of the main features of this study. Note: Marks the distribution of the subjects (sessions) and their correlations (regression lines) for each combination of the features. The diagonal cells describe the overall frequency distribution of each group. Orange indicates patients and blue indicates healthy controls. Green lines indicate regression lines with shaded areas indicating confidence band of the regression lines.

Figure 2 illustrates the distributions and intercorrelations of the features used in this study. Generally, the collinearity between variables was not very high. Rather, VAS exhibited significant negative correlations with SCR peak (*r* = −0.163, *p* = 0.005) and SCR amplitude (*r* = −0.174, *p* = 0.003), indicating that blunt SCRs were characteristic of more anxious participants in this study. Strong associations between the subfactors of HRV and SCR were observed: *r* = 0.422 between SDNN and LF/HF ratio; *r* = 0.296 between SCR peak and amplitude; *p*s < 0.001. In contrast, HR exhibited no correlation with LF/HF ratio (*r* = 0.034) and was even negatively correlated with SDNN (*r* = −0.245, *p* < 0.001). SCR peak showed positive correlations with SDNN (*r* = 0.219, *p* < 0.001) and LF/HF ratio (*r* = 0.141, *p* = 0.015), despite negative correlations with VAS, possibly reflecting a general arousal response that may be independent of anxiety or negative affect.

### 3.2. Overall Performance and Robustness

Table 2 summarizes the overall performance of the classification models. All models achieved accuracy, specificity, sensitivity, and precision scores of around 0.80 except for k-NN. Although the training process involved threshold tuning based on the F1 score that focuses on sensitivity (i.e., the proportion of actual patients correctly classified as patients) and precision (i.e., the proportion of the predicted patients who were truly patients), most models reached high overall performance including specificity (i.e., the proportion of healthy participants correctly classified as healthy) scores. Accuracy and specificity scores were the highest for LRR (0.83–0.86) but the lowest for k-NN (below 0.80). Sensitivity was higher for SVC and ensemble models such as RF and SGB, all of which exceeding 0.82, compared with baseline or linear models.

The performance scores across various machine learning models underscore the feasibility and potential clinical utility of this VR-based diagnostic framework for panic disorder and agoraphobia. The sensitivity scores of up to 0.83 suggest that the system reliably detects symptomatic individuals, reducing the likelihood of false negatives that could delay timely intervention. The consistently strong accuracy, specificity, and precision indicate balanced classification, limiting unnecessary follow-ups for healthy participants. Moreover, the robust performance across diverse hyperparameter settings suggests the framework’s potential generalizability to different samples, enhancing its prospects for real-world deployment in screening or stepped-care pathways.

### 3.3. Subgroup Analysis

**Figure 3:**
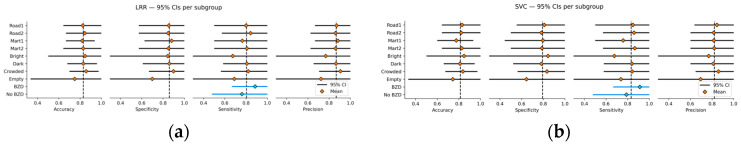
Forest plots of subgroup performance of Logistic Ridge Regression (**a**) and *C*-Support Vector Machine (**b**) models (*C* = 1 for each model). Note: Each data point reflects the total distribution of performance scores across 1000 iterations of train-test splits conducted within the indicated subgroup: VR scenes (Road 1, Road 2, Mart 1, Mart 2), brightness (bright, dark), crowd density (crowded, empty), and concomitant benzodiazepine (BZD) use. BZD subgroups are based on the patient data, and other subgroups include the entire session data. The vertical dotted lines represent the mean scores across all participants. Diamond markers denote the mean performance across the 1000 splits and horizontal lines indicate 95% confidence intervals (CIs). Lines and diamond markers for BZD subgroups were highlighted in blue.

Figure 3 presents subgroup performance scores for LRR and SVC models across various conditions including VR scenes, personalized VR settings, and concomitant BZD usage. Although direct comparisons were difficult due to the limited sample sizes of each subgroup, the performance scores generally remained consistent across subgroups, with no substantial differences observed. Furthermore, the models demonstrated adequate sensitivity regardless of BZD intake, indicating reliable symptom prediction irrespective of medication status. The minimal variability in performance across subgroups highlights the robustness of the models and supports their potential generalizability to diverse VR scenarios and contexts. These findings also imply that the models can capture distinct behavioral or physiological response patterns in patients with panic disorder and agoraphobia, even in the presence of psychotropic medication effects.

### 3.4. Feature Importance

**Figure 4:**
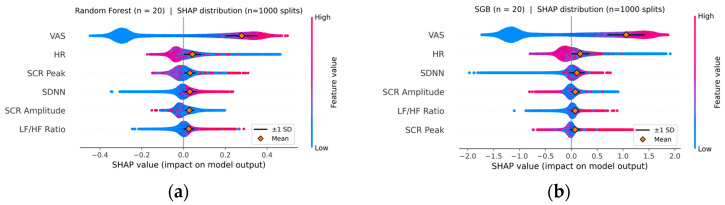
SHAP summary plots for the Random Forest (**a**) and Stochastic Gradient Boosting (**b**) models (*n* = 20 for each). Note: Feature importance was determined using the Shapley Additive Explanation (SHAP) values derived from 1000 iterations of train-test splits. Each violin plot shows the distribution of SHAP values for each feature across splits. Orange diamond markers denote the mean absolute SHAP values, and navy horizontal lines indicate standard deviations.

Figure 4 demonstrates feature importance as determined by SHAP values for RF and SGB models. For both models, VAS exhibited substantially higher SHAP magnitude than the other features, followed by HR. It is notable that HR exhibited relatively high importance compared to other physiological features, even though the between-group difference in HR was not significant in the traditional *t*-test (*p* = 0.915). Among the physiological variables, HRV features demonstrated greater importance than SCR features, although in traditional statistics, SCR amplitude showed the largest raw group difference (*p* = 0.003). In short, SHAP highlights complex interactions or non-linear relationships captured by ensemble models that classical univariate tests may fail to detect.

One illustration of such relationships can be the interaction between SCR amplitude and the change in state anxiety following VR exposure in patients versus healthy people. In this study, whereas healthy participants exhibited a positive correlation between the increase in SCR amplitude and the rise in STAI-S scores after VR exposure compared to baseline (*r* = 0.229, *p* = 0.005), patients’ increase in STAI-S was associated with lower SCR amplitude (*r* = −0.253, *p* = 0.002). This relationship persisted even after the effect of BZD medication was controlled for (partial *r* = −0.242, *p* = 0.003), highlighting the relevance of blunted SCRs as a marker for distinguishing patients from healthy individuals.

## 4. Discussion

This study is a pioneering work that combines VR technology and physiological measurements to potentially diagnose panic and agoraphobia symptoms. This study analyzed self-reports and physiological responses collected from patients and healthy people during the VR exposure, yielding promising results. The identified models showed high overall classification performance, maintaining robustness across VR environments, personalized exposure settings, medication status, and model tuning conditions. Although the self-reported VAS exhibited the highest predictive ability, the findings also highlight the clinical relevance of physiological markers in panic and agoraphobia, particularly the attenuated SCR amplitude responses observed in patients. Moreover, through the nested threshold tuning optimized for the F1 score, this study achieved sensitivity scores above 80%, indicating promise for the clinical utility of this approach in screening patients with panic disorder and agoraphobia.

To our knowledge, this is the first study to attempt classification of panic and agoraphobia symptoms based on physiological data collected with VR stimuli as an umbrella model. The current findings indicate that the VR exposure proposed in this study can be a valid diagnostic tool for panic disorder and agoraphobia. The incorporation of VR-based methods offers substantial advantages over traditional assessment approaches, particularly by enhancing accessibility for patients and improving cost-effectiveness [2,20]. VR tools can also provide rich, ecologically valid assessment contexts by capturing patients’ in situ responses, facilitating seamless integration between diagnostic and treatment environments [21].

Looking at the differences in the features between patients and healthy people, VAS demonstrated the highest differences overall, both in traditional and machine learning analyses, which is consistent with the previous findings [4]. However, as emphasized in the Research Domain Criteria (RDoC) framework proposed by the United States National Institute of Mental Health, self-report and behavioral assessments should be adequately supplemented by objective measurements such as neurophysiological indicators [21,22]. As also shown in this study, patients experiencing panic and agoraphobia may report a wide range of VAS scores, with some individuals not expressing significant anxiety. Moreover, as discussed above, self-reported anxiety may not be a feature that is specific to panic and agoraphobia, even if they are measured along with agoraphobic stimuli. For these reasons, adding various physiological predictors and establishing precise models for diagnosis and assessment are needed, based on multimodal principles, although subjective anxiety plays a most important role in the diagnosis of panic disorder and agoraphobia.

On the contrary, although HRV features seem to be valid predictors in the machine learning models, their predictive ability was not clearly demonstrated in traditional statistics. Such results are assumed to have stemmed from the offset between the arousal responses and their low stress resistance. Although the HRV features of the patients increased because the VR stimuli induced anxiety only among the patients, the differences in the magnitude may have shrunk due to their low responsiveness to stress and the concomitant bluntness of the responses [9]. In fact, some studies reported that under the VR or computer stimuli, patients with panic disorder and agoraphobia exhibit ambivalent reactions characterized by either blunt or overreactive responses [11,15]. Therefore, besides the establishment of the model to detect such ambivalent responses, an adequately adjusted and, if needed, personalized VR exposure program is required to elicit the optimized reactions of the patients, encompassing both blunt and overreactive responses. This includes settings that reflect the biological reactivity of the patients, encompassing the length of exposure sessions, difficulty of exposure, and tailored settings for each patient, as discussed further below.

Regarding the SCR features, although they seem to be valid for the diagnosis, further studies are needed to elucidate their significance. Although SCR amplitude exhibited strong group differences in the traditional statistics, its direction, which was lower for the patient group, was inconsistent with previous observations [11,14]. Overall, the SCR patterns observed in patients with panic disorder and agoraphobia in this study also indicated blunted physiological reactivity, as shown in the interaction effect between patients and healthy individuals in the relationship between state anxiety and SCR amplitude. Similar patterns were reported among some patients with anxiety disorders in a previous study [23]. Indeed, blunted physiological reactivity is not uncommon among patients with heightened anxiety [24]. In particular, chronic anxiety may result in sympathetic overactivation, which can paradoxically reduce physiological responsiveness in stress-related contexts among patients with panic and agoraphobia [25,26]. Acute overactivation and hyperarousal frequently culminate in neural exhaustion, which may also contribute to dysregulation in fear circuitry in pervasive anxiety [26,27]. Agoraphobia is frequently characterized by a chronic course [28,29]. However, in the present study, the chronicity of the patients’ conditions was not thoroughly examined, calling for future research to address illness duration and its neurophysiological correlates in greater depth.

Such an issue may also be attributable to the relatively low anxiety-inducing settings employed in this study. In our VR exposure program, the training difficulty can be modulated by adjusting the length of exposure time or the occurrence of various random events, such as a traffic accident on the road or a fire at the supermarket. Nevertheless, in this pilot study, only a limited number of simple events were presented within relatively brief sessions, which can also explain why the VAS scores of the patients were not that high. This approach was intentionally designed to create a safe and controlled exposure environment, thereby minimizing the risk of inducing severe panic attacks while maintaining the feasibility of applying it in a general clinical setting. Nevertheless, relatively mild stimuli or threat cues may elicit attenuated responses in both self-reported and physiological measures [27,30]. To address this limitation, we are conducting a follow-up study that leverages this adaptability feature for intervention purposes. The validity of personalized exposure has already been demonstrated in our previous findings [16].

Another limitation of this study is that although we conducted subgroup analyses addressing between-condition differences across VR environments and settings, we did not take into account the within-session or within-subject differences (i.e., the changes in self-reported or physiological responses over time/session). In this study, physiological measures such as SDNN, SCR peak, and SCR amplitude exhibited an increasing trend across sessions (*p*s = 0.001–0.005 for the correlations with the session number). Also, the generalized estimating equation model revealed that the magnitude of within-session changes in VAS (*p* < 0.001) and SCR amplitude (*p* = 0.026) had differences between patients and healthy people, indicating that over the session, VAS scores tended to increase more among patients than among the healthy people, whereas SCR amplitude tended to increase more among the healthy people than among patients. However, these effects were ignored in this study to simplify the model structure. Moreover, we only used the session-wide data without focusing on the specific time points during the sessions when the important stimuli were presented. Multiple studies suggest that incorporating real-time, dynamic stimuli or applying event-based analyses such as EEG event-related potentials can greatly enrich VR assessment and diagnostic frameworks [31,32,33]. Further studies may consider adopting event-related responses by synchronizing the specific VR events with physiological signals. We hope that this work will provide insights into data-driven research in VR-based assessment using physiological measures and facilitate future discovery of potential biomarkers for symptoms of panic disorder and agoraphobia.

Although this study provides meaningful insights into the potential use of VR technology and physiological measures in diagnosing panic disorder and agoraphobia, the findings should be considered preliminary. Further validation through external datasets and diverse patient populations is essential to ensure generalizability and robustness. Future research should aim to replicate and extend these findings, ultimately confirming the clinical utility and reliability of VR-based multimodal assessment approaches for panic and agoraphobia symptoms.

## 5. Conclusions

Our findings indicate that VR exposure combined with physiological and subjective measures holds promise as a diagnostic approach for panic disorder and agoraphobia, which may contribute to the advancement of data-driven assessment strategies for anxiety disorders. Further validation using diverse populations and event-related analyses will ensure the reliability and clinical utility of this multimodal machine learning framework.

## Figures and Tables

**Table 1:** Descriptive statistics and group differences based on traditional statistics.

	PD/A Patients (*n* = 38, Sessions = 148)	Healthy Control (*n* = 38, Sessions = 147)	*p* for *t*/χ^2^
Demographics and baseline characteristics			
Sex (female)	23 (61%)	21 (55%)	0.810
Age	30.39 ± 13.15	27.45 ± 11.28	0.298
Education (years)	13.97 ± 2.17	14.32 ± 1.80	0.450
PDSS (baseline)	7.95 ± 5.13	0.08 ± 0.36	**<0.001**
STAI-S (baseline)	55.83 ± 12.42	33.82 ± 8.18	**<0.001**
STAI-S (after exposure)	53.32 ± 12.58	33.87 ± 8.09	**<0.001**
Antidepressants	14 (37%)	0 (0%)	**<0.001**
BZDs	13 (34%)	0 (0%)	**<0.001**
Features			
VAS	2.67 ± 1.86	0.30 ± 0.68	**<0.001**
HR	78.56 ± 12.22	78.69 ± 7.12	0.915
SDNN	97.40 ± 86.82	80.75 ± 47.51	**0.042**
LF/HF ratio	1.24 ± 1.87	1.02 ± 1.20	0.235
SCR peak	9.66 ± 11.95	11.25 ± 9.10	0.199
SCR amplitude	0.080 ± 0.102	0.151 ± 0.266	**0.003**

Note: The demographics and baseline characteristics were based on the participant data. The feature statistics were based on the session-wide data. The bold numbers indicate statistically significant *p*-values (<0.05). Abbreviations: PD/A, panic disorder or agoraphobia; PDSS, Panic Disorder Se-verity Scale; STAI-S, State-Trait Anxiety Inventory–State; BZD, benzodiazepine; VAS, Visual An-alogue Scale for current anxiety level; HR, heart rate; SDNN, standard deviation of nor-mal-to-normal interval; LF/HF, low frequency divided by high frequency; SCR, skin conductance response.

**Table 2:** The overall performance scores for the machine learning models.

Models	Accuracy [95% CI]	Specificity [95% CI]	Sensitivity [95% CI]	Precision [95% CI]
Gaussian Naïve Bayes				
	0.797 [0.661–0.914]	0.808 [0.556–1.00]	0.787 [0.591–0.936]	0.823 [0.636–1.00]
*k*-Nearest Neighbors				
*k* = 1	0.725 [0.596–0.845]	0.760 [0.548–0.929]	0.694 [0.486–0.900]	0.762 [0.588–0.917]
*k* = 3	0.740 [0.610–0.862]	0.757 [0.429–0.964]	0.729 [0.471–0.968]	0.778 [0.578–0.960]
*k* = 5	0.751 [0.614–0.879]	0.733 [0.470–1.00]	0.771 [0.467–0.968]	0.767 [0.583–1.00]
*k* = 7	0.768 [0.621–0.898]	0.772 [0.500–1.00]	0.765 [0.484–0.967]	0.793 [0.613–1.00]
*k* = 9	0.771 [0.627–0.897]	0.789 [0.519–1.00]	0.756 [0.516–0.963]	0.805 [0.615–1.00]
Logistic Ridge Regression				
*C* = 0.1	0.831 [0.690–0.948]	0.862 [0.633–1.00]	0.802 [0.600–0.964]	0.870 [0.687–1.00]
*C* = 1	0.830 [0.690–0.948]	0.858 [0.640–1.00]	0.804 [0.594–0.964]	0.866 [0.676–1.00]
*C* = 3	0.829 [0.690–0.947]	0.856 [0.630–1.00]	0.805 [0.600–0.964]	0.865 [0.676–1.00]
*C* = 6	0.829 [0.693–0.948]	0.857 [0.630–1.00]	0.804 [0.586–0.964]	0.865 [0.679–1.00]
*C* = 10	0.829 [0.695–0.948]	0.857 [0.654–1.00]	0.804 [0.600–0.964]	0.865 [0.679–1.00]
*C*-Support Vector Machine				
*C* = 0.1	0.779 [0.618–0.914]	0.722 [0.429–0.964]	0.833 [0.576–1.00]	0.771 [0.585–0.964]
*C* = 1	0.813 [0.678–0.931]	0.794 [0.571–1.00]	0.832 [0.583–0.969]	0.821 [0.643–1.00]
*C* = 3	0.807 [0.672–0.917]	0.795 [0.543–1.00]	0.818 [0.586–0.968]	0.820 [0.636–1.00]
*C* = 6	0.797 [0.655–0.914]	0.786 [0.538–1.00]	0.809 [0.571–0.968]	0.811 [0.636–1.00]
*C* = 10	0.784 [0.649–0.900]	0.775 [0.514–1.00]	0.793 [0.548–0.967]	0.800 [0.621–1.00]
Random Forest				
*n* = 10	0.804 [0.667–0.929]	0.780 [0.500–1.00]	0.827 [0.613–0.968]	0.812 [0.630–1.00]
*n* = 20	0.817 [0.677–0.932]	0.802 [0.548–1.00]	0.831 [0.633–0.968]	0.827 [0.636–1.00]
*n* = 30	0.819 [0.678–0.932]	0.805 [0.548–1.00]	0.833 [0.645–0.968]	0.830 [0.636–1.00]
*n* = 50	0.817 [0.678–0.933]	0.801 [0.548–1.00]	0.833 [0.633–0.968]	0.827 [0.634–1.00]
*n* = 70	0.819 [0.679–0.933]	0.802 [0.538–1.00]	0.834 [0.633–0.968]	0.828 [0.644–1.00]
*n* = 90	0.820 [0.679–0.933]	0.806 [0.556–1.00]	0.833 [0.636–0.968]	0.831 [0.655–1.00]
Stochastic Gradient Boosting				
*n* = 10	0.818 [0.677–0.932]	0.817 [0.567–1.00]	0.820 [0.600–0.968]	0.837 [0.636–1.00]
*n* = 20	0.819 [0.679–0.931]	0.817 [0.571–1.00]	0.821 [0.625–0.968]	0.838 [0.655–1.00]
*n* = 30	0.816 [0.684–0.915]	0.818 [0.593–1.00]	0.815 [0.607–0.967]	0.837 [0.645–1.00]
*n* = 50	0.810 [0.672–0.919]	0.821 [0.577–1.00]	0.801 [0.600–0.966]	0.836 [0.645–1.00]
*n* = 70	0.808 [0.677–0.921]	0.823 [0.593–1.00]	0.794 [0.567–0.967]	0.837 [0.643–1.00]
*n* = 90	0.805 [0.672–0.917]	0.823 [0.592–1.00]	0.789 [0.559–0.964]	0.835 [0.647–1.00]

Note: The scores are the average performance from 1000 iterations of the stratified train-test splits. Abbreviations: CI, confidence interval; *k*, number of neighbors; *C*, inverse of the strength of regularization; *n*, number of estimators.

## Data Availability

The datasets generated and analyzed during the current study are not publicly available due to privacy and ethical restrictions but are available from the corresponding author on reasonable request.
